# Genetic Analysis of the Atrial Natriuretic Peptide Gene Polymorphisms among Essential Hypertensive Patients in Malaysia

**DOI:** 10.1155/2016/6712529

**Published:** 2016-06-20

**Authors:** Nooshin Ghodsian, Patimah Ismail, Salma Ahmadloo, Narges Eskandarian, Ali Etemad

**Affiliations:** Genetic Research Group, Department of Biomedical Science, Faculty of Medicine and Health Sciences, Universiti Putra Malaysia, 43400 Serdang, Selangor, Malaysia

## Abstract

*Background*. Atrial natriuretic peptide (*ANP*) considerably influences blood pressure regulation through water and sodium homoeostasis. Several of the studies have utilized anonymous genetic polymorphic markers and made inconsequent claims about the* ANP *relevant disorders. Thus, we screened Insertion/Deletion (ID) and G191A polymorphisms of* ANP *to discover sequence variations with potential functional significance and to specify the linkage disequilibrium pattern between polymorphisms. The relationships of detected polymorphisms with EH with or without Type 2 Diabetes Mellitus (T2DM) status were tested subsequently.* Method*.* ANP *gene polymorphisms (*I/D *and* A191G*) were specified utilizing mutagenically separated Polymerase Chain Reaction (PCR) in 320 subjects including 163 EH case subjects and 157 controls.* Result*. This case-control study discovered a significant association between* I/D *polymorphisms of* ANP *gene in EH patient without T2DM. However, the study determined no association between G191A polymorphisms of* ANP *in EH with or without T2DM. In addition, sociodemographic factors in the case and healthy subjects exhibited strong differences (*P* < 0.05).* Conclusion*. As a risk factor,* ANP *gene polymorphisms may affect hypertension. Despite the small sample size in this study, it is the first research assessing the* ANP *gene polymorphisms in both EH and T2DM patients among Malaysian population.

## 1. Introduction

Hypertension, known as high blood pressure or a silent killer, is classified into 2 types: primary (essential) and secondary (pulmonary) form of hypertension. Essential hypertension (EHT) accounts for about 95% of people with hypertension and is used to describe hypertension when no specific cause is found according to the World Health Organization [1]. Hypertension is a significant public health issue for its damaging consequences globally [2]. According to World Health Organization, hypertension is widespread in plenty of developing economies. Globally, 1.56 billion people (29%) of adult population are predicted to suffer from hypertension by the year of 2025. Nearly seven million deaths annually might be affected by hypertension as stated by Singh et al. [3]. The heightened risk of stroke, kidney disease, heart attack, and heart failure will result as the blood pressure increases. In another study by Chobanian et al. [4], several risk factors (e.g., diabetes, high cholesterol levels) also raise the CVD risk from hypertension.

Previous prospective and case control studies have shown that hypertension progression is an independent predictor of type 2 diabetes [5]. Several possible factors are likely to be causes of the association between Type 2 Diabetes Mellitus (T2DM) and hypertension. The relationships between hypertension and diabetes were also obtained by other research groups [6]. Considering the fact that 88 individuals out of 163 hypertension cases suffered from diabetes, a strong relation was established between hypertension and diabetes in the study.

Genetics is claimed to contribute to hypertension. Genetic evidence influencing blood pressure comes from various sources. Lifton et al. [7] mention that high and low blood pressure, as one of rare Mendelian forms, are considerably influenced by single genes. The genetic influence or heritability estimation on blood pressure (BP) variation displays remarkable range (30 to 50%) [8]. Genetic variations can considerably affect EHT genesis which significantly exhibits risk factor for progressive renal damage, stroke, ischemic heart disease, and peripheral vascular disease [9]. Most recent studies have conducted an investigation of genetic causes of essential hypertension associated with analysis of candidate genes. The investigated candidate genes include the following.

Atrial natriuretic peptide (*ANP*) is located on chromosome 1p36.2. Its main product (atrial natriuretic peptide protein) acts as cardiac hormone which is synthesized substantially within the heart and stored in the atrial myocyte as prohormones for rapid release in response to stimuli [10].* ANP* is a 28-amino acid peptide with a 17-amino acid ring in the middle of the molecule. The ring is formed by a disulfide bond between two cysteine residues at positions 7 and 23 [11]. The heart is considerably affected by salt and water balance regulation as reported by Lee and Burnett [12]. Atrial natriuretic peptide (*ANP*) receptor, as a cardiac hormone, is involved in the physiological maintenance of blood volume and arterial blood pressure [13].

Insertion/deletion (*I*/*D*) polymorphism, which is 8-bp biallelic, is located in the second intron of the human atrial natriuretic peptide gene. The deletion variant might participate in the functional impairment of natriuretic peptide system defining an increased genetic susceptibility to hypertension [14].

The G191A polymorphism, which appears similarly in the previous studies as G664A but is used as G191A polymorphism and is mapped in a hydrophobic leader segment, is removed from the mature* ANP* (exon 1). In recent studies on G191A polymorphism, strong association with the cardiovascular disorders (including hypertension) was reported. The results with null findings were obtained for G664A including hypertension and other cardiovascular disorders [15]. However, the other studies indicated a significant association between G191A and hypertension in black Africans with the positive results for* Hpa* II RFLP [16]. Previous studies reported the association of* A191G* polymorphisms in European Americans and in black Africans, which was detected across the three ethnic groups but not for Japanese population. Given these findings, it will be of interest to investigate whether some of the ANP polymorphisms reported to date have arisen after human population differentiation [15].

Based on literature review, most of the studies were done on ANP gene polymorphisms associated with EHT (but their interactions within T2DM were not investigated). So this study was the first research conducted to determine genetic polymorphism of the I/D and G191A among EHT subjects with or without T2DM in Malaysian subjects.

## 2. Material and Method

### 2.1. Study Subject

In the current study, 163 Malaysian case subjects and 157 controls were analyzed. The controls were recruited based on the following criteria: (1) no history of EHT and Type 2 Diabetes Mellitus; (2) Systolic Blood Pressure (SBP) ≤ 140 mm Hg and DBP ≤ 90 mm Hg as measured with a digital sphygmomanometer; and (3) no recent symptoms of heart and renal disorders. The case subjects were recruited based on the following criteria: EHT history; SBP > 140 mm Hg and/or Diastolic Blood Pressure (DBP) > 90 mm Hg measured by a digital sphygmomanometer; and biological or clinical signs of pulmonary hypertension. Controls were selected from 168 consecutive volunteers without EHT histories. Among these, 11 subjects were eliminated for missing DNA extraction. The case subjects were selected in the Seremban Hospital. Between December 2011 and June 2012, we specified 168 patients of whom 5 subjects were excluded because they did not fit the blood pressure criterion. Ethical approval was acquired from Universiti Putra Malaysia and Seremban Hospital. All participants were asked to fill in informed consent questionnaires. The samples were used with reference number UPM.FPSK.PADS/T7-MJETIKAPer/F01-JSB-Mac.

### 2.2. Measurement

Body mass index (BMI) was calculated by measuring of case and control subjects' height and weight. Blood pressure was evaluated by measuring SBP and DBP with a safe, reproducible, accurate, and noninvasive method to screen Malaysian populations.

### 2.3. Biochemical Analysis

The mean of three consecutive measurements was computed. Plasma was extracted for determination of DNA extraction and standard biochemical measurements at the end of this procedure. Peripheral venous blood samples were collected after an overnight fasting in control subjects who were participating. In this section, serum electrolytes were utilized to examine the lipid profiles which included triglycerides (TG), total cholesterol (TCH), low-density lipoprotein (LDL), and high-density lipoprotein (HDL). Fasting Blood Sugar (FBS) is also measured with standard laboratory techniques. It was noticeable that we had referred to the hospital to assess biochemical information for cases' documents.

### 2.4. Genotype Investigations

In the study, the buccal and blood cells were collected from study group (hypertensive patients) and controls, respectively. The blood was kept in ethylenediaminetetraacetic acid (EDTA) tube and stored at 4°C for a maximum of three days before utilizing. The DNA was applied for amplification after extracting from buccal and blood cell samples using Qiagen Kit (Germany); then, it was stored at −20°C for later usage. DNA was qualified right after all primers were optimized by PCR method. By utilizing the nanodrop in two optical density (OD) wave lengths (260 nm and 280 nm), the extracted DNA concentration was examined.

Genomic DNA was amplified by multiplex-PCRs. Utilizing the mutagenically separated PCR technique, I/D (in intron 2) and the G191A polymorphisms (in exon 1) were genotyped. Each reaction was composed of 6x master mix (including DNA polymerase, MgCl_2_, dNTPs, and reaction buffers), 0.6 *μ*L relative primers (0.3 *μ*L forward and 0.3 *μ*L reverse), and 1 *μ*L of genomic DNA and ultimately distilled water was added to a final volume of 25 *μ*L. For each polymorphism, several PCR and DNA amplifications were carried out with forward and reverse primers at the specific temperatures in several conditions (Table 1). Amplified PCR products were analyzed by gel electrophoresis methods with agarose.

### 2.5. Sequencing

In order to confirm the genotyping results, random samples were used and repeated with the same PCR conditions. To receive a final confirmation of the nucleotide sequences, purified PCR products were sent to Research Biolabs Malaysia. The sequencing results were aligned with the gene sequence from the NCBI-GenBank by the MEGA4 software.

### 2.6. Statistical Analysis

The statistical analysis in the present study was conducted by statistical package for the social science (SPSS version 22). Utilizing two-tailed Student's *t*-test and one-way ANOVA test, all variables among the groups and the group means were being contrasted (*P* < 0.05 was viewed to be significant statistically). The distribution of genotypes with Hardy-Weinberg expectations was calculated using a chi-squared test. Allelic frequencies were analyzed by gene-counting method. In order to detect the effects of high risk alleles, odds ratios (OR) with 95% confidence intervals (CI) were checked as well.

## 3. Result

### 3.1. Sociodemographic Factors

In this study, three different races' population were selected for our searching program including Malay, Chinese, and Indian subjects. It was also divided into three groups including EHT (*n* = 75), EHT + T2DM (*n* = 88), and control group (*n* = 157). The associations of clinical characteristics as major risk factors with ANP gene polymorphisms were investigated among EHT subjects with or without T2DM in Malaysia.

The mean and the standard deviation (SD) of the clinical characteristics were clearly indicated in Table 2. The mean age of EHT and EHT + T2DM subjects was nearly equal (59.45 ± 10.34, 59.05 ± 11.10) but higher compared to the controls (52.51 ± 9.41). Considering age, Systolic Blood Pressure (SBP), Diastolic Blood Pressure (DBP), Body Mass Index (BMI), Fasting Blood Sugar (FBS), low-density lipoprotein (LDL), and triglyceride (TG), the subjects' clinical characteristics illustrated significant differences for mentioned parameters between hypertensive and normotensive subjects (*P* < 0.05). Noticeably, EHT subjects showed the major amount of SBP and DBP (152.01 ± 23.10, 94.12 ± 10.00). Besides, EHT + T2DM subjects showed the major amount in BMI and FBS which were 28.04 ± 4.25 and 7.89 ± 1.35, respectively. The amount of TCH and HDL did not show any significant differences between EHT and control subjects; however, EHT + T2DM subjects maintained minimum record of TCH (4.53 ± 1.04) in comparison with controls. Besides, LDL (3.21 ± 1.10) in controls' mean displayed the highest parameter in contrast to other groups. According to Table 2, the significant difference was obtained in the level of age (*P* value = 0.000), SBP (*P* value = 0.000), DBP (*P* value = 0.000), BMI (*P* value = 0.000), FBS (*P* value = 0.000), and TG (*P* value = 0.000) between EHT subjects with or without T2DM and control.

### 3.2. *I/D*

**Figure 1:**
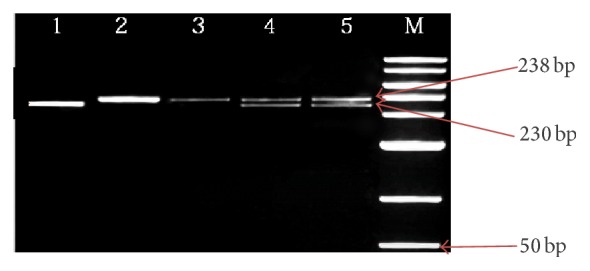
I/D polymorphism detection of ANP gene in 5% agarose gel electrophoresis. The picture represents homozygote (DD) in lane 1; lanes 2 and 3 show homozygote (II) and lane 4 represents heterozygote (I/D). Lane 5 genotype has been used as a control and to determine I/D. M represents a 50 bp DNA Ladder Plus (Bioline).

Analysis of* ANP*'s* I*/*D* mutation with 230 and 238 bp PCR product with mutation (I to D) was amplified by PCR. The* I*/*D* mutation of* ANP* gene was detected using 5% agarose gel electrophoresis.  Figure 1 displays three different bands referring to three different genotypes of* I*/*D* polymorphism (II, ID, and DD). To analyze the* I*/*D* variation of* ANP* gene, we contrasted the EHT subjects with or without T2DM to the controls.

Table 3 shows the distribution of* I*/*D* polymorphic genotypes and allele frequencies of* ANP* gene between EHT, EHT with diabetes, and control subjects. There was a significant value of genotype (*P* = 0.027) and allele frequencies (*P* = 0.015) between EHT subjects and controls (*P* < 0.05). There was no significant difference between EHT + T2DM subjects and controls (*P* value > 0.05). After post hoc test, neither genotype nor allele frequencies showed association between EHT subjects and controls. No significant value was achieved between EHT + T2DM subjects and controls.

### 3.3. *A191G*

**Figure 2:**
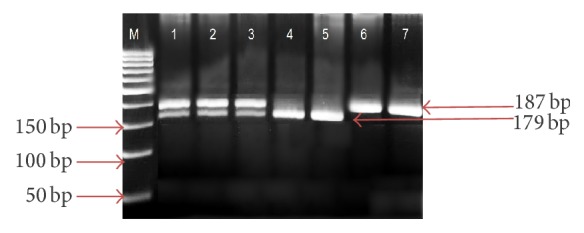
PCR product amplification of* ANP* gene in 5% agarose gel electrophoresis. The figure demonstrates the PCR product amplification of* A191G* polymorphism of* ANP *gene. Lanes 1, 2, and 3 show GA (189 and 197 bp); lanes 4 and 5 show GG; and lanes 6 and 7 show AA. M represents a 50 bp DNA Ladder Plus (Bioline).

Analysis of* ANP*'s* A191G* polymorphism with 189 and 197 bp PCR product with mutation (G to A) was amplified by PCR. In Figure 2, analysis of* A191G* polymorphism amplification product displayed three different variations (GG, GA, and AA) in* A191G* polymorphism.

The distribution of* A191G* polymorphic genotypes of* ANP* gene between the EHT, EHT + T2DM, and control subjects is indicated in Table 3. No significant difference was obtained in genotypes and allele frequencies between EHT patients and control group with *P* value of 0.761 and 0.067, respectively. Moreover, there were no significant differences of genotype and allele frequencies between EHT + T2DM subjects and control group which were 0.451 and 0.534, respectively.

### 3.4. Genotype Analysis Based on Race

As this study is a heterogeneous research, race was examined to specify for any significant role among genotypes. The genotypes of each groups based on race were analyzed in Table 4. Analyzing the ID genotypes base on race demonstrated significant difference between genotypes in EHT subjects (*P* < 0.033); besides, no significant differences were achieved among genotypes in EHT + T2DM subjects and/or in controls. Significant differences* were* observed between race and genotype of A191G in two groups which were EHT subjects (0.046) and control (0.021). However, no significant difference was observed between race and genotype in EHT + T2DM subjects in this table.

### 3.5. Allele Frequency Analysis Based on Gender

Regarding the importance of gender in the study, gender and allele frequency for* I*/*D* polymorphism were analyzed among EHT subjects with or without T2DM.  Table 5 indicates the allele frequencies in each of the three groups. No significant differences were observed between the gender and each polymorphism.

## 4. Discussion

The aim of the present case-control study was to evaluate the effect of two polymorphism variants (*I*/*D* and* G191A*) on elements of the atrial natriuretic peptide and blood pressure. Blood pressure was evaluated by measuring SBP and DBP with a safe, reproducible, accurate, and noninvasive method to screen Malaysian populations. The study affirms that blood pressure and age are major determinants of hypertension. Negative association was achieved between EHT and HDL or TCH in both case and control subjects in the study; however, findings were also inconsistent in some other populations in the other studies. These discrepancies may be explained by several factors: the population under study may be affected by the sex and age distributions. The other possibility is that these inconsistencies might be the physiological mechanisms' results which act distinctly depending on sex and age.

High blood pressure is an important risk factor for CVD that is 26.4% around the world and it is increasing up to 29.2% in 2025. According to NHANES, the percentage is about 29.3% in United States (2003-2004). In Malaysia, the prevalence of EHT was about 32.2% in 2006 among the elderly. However, the prevalence of hypertension in the present study is 50.90%, which is higher in comparison to the study conducted by NHMS [17].

In many instances, genetic, environmental, and demographic complicated interactions lead to hypertension. Older age group is more likely to develop hypertension. Besides, it tends to increase rapidly with aging. Camm [18] also states that blood pressure increases with age, except where salt intake is low, physical activity is high, and obesity is not present. In this study, we have found a significant difference in hypertensive (where the hypertensive age was higher with *P* < 0.05) in comparison to normotensive subjects.

Significant differences in age, SBP, DBP, BMI, FBS, LDL, and TG were found between hypertensive and normotensive subjects (*P* < 0.05), although HDL and TCH levels showed no significant differences in hypertensive and controls (*P* > 0.05). Significant differences of clinical characteristics (BMI, HDL, LDL, TG, TCH, SBP, and DBP) were identified between healthy individuals and EHT subjects as reported previously. Considerable variations were observed in triglycerides and total cholesterol between the groups similar to Asian Indian population study [19]. However, the risk factors (e.g., DBP, LDL, and HDL) have not shown any significant difference between case and control results in previous study of Asian Indian population. According to the finding, the increase of triglycerides and HDL reduction increase the chance of initial development and occurrence of the disease [20]. Hence, we were required identifying the etiological factors associated with hypertension disorders among the Malaysian population.

Gene searches, which affect the primary hypertension development in population, resulted from genetic analysis effective techniques (particularly genome-wide linkage analysis). Hsueh et al. [21] state that fundamental links of blood pressure to many chromosomal regions (as regions linked to familial combined hyperlipidemia) have been statistically identified by technique applications. Thus, there are several genetic loci that influence blood pressure in general population according to these findings. Nevertheless, Harrap et al. [22] studies indicate that recognizable single genes which affect hypertension are found to be unusual and match up with a multifactorial cause of hypertension. The results of Dominiczak et al. [23] studies showed relation between genes and blood pressure changes in 50% of cases.

The aim of the present case-control study was to evaluate the effect of two polymorphism variants (I/D and G191A) on elements of the atrial natriuretic peptide in hypertension to specify potential association affecting high blood pressure determination. The study affirms that blood pressure and age are major determinants of hypertension.

### 4.1. *ANP I/D* Polymorphism

The natriuretic peptides (specifically* ANP*) are increasingly recognized to play a fundamental role in BP regulation [24]. The role in BP regulation reflects the pluripotent cardiorenal actions of* ANP*, which include diuresis, enhancement of renal blood flow and glomerular filtration rate, systemic vasodilatation, suppression of aldosterone, and inhibition of the sympathetic nervous system. In addition to recent human studies, these actions of* ANP* demonstrate an association between higher plasma of* ANP* and a lower risk of EH [25].

Notably, strong association was observed between the* ANP I/D* polymorphism and hypertension in current study. The existence of the* ANP* D allele indicated relationship with increased SBP and age in both genders in the hypertensive subjects. The fact that the* ANP* polymorphism is related to SBP (merely in case) may be related to a hormone of atrial natriuretic peptide influences on the existence of hypertension.

In a previous study [26], the* ANP* system genetic variants are involved in the EHT etiology.* ANP*, which is in response to increased blood volume, acts to reduce water, sodium, and adipose loads on the circulatory system, thereby reducing blood pressure. It is mainly synthesized by atrial myocytes and released by relaxing blood vessels, where it acts by reducing peripheral resistance, increasing the glomerular filtration rate inhibiting renin release. It causes significant increases in urinary sodium excretion and urine output; this mechanism is involved in the regulation of BP [25].

### 4.2. *ANP G191A* Polymorphism

In humans, different single nucleotide polymorphisms (SNPs) have been identified in* ANP* gene. They seem to be in relation to high blood pressure and low plasma* ANP* levels and leave ventricular hypertrophy in subjects. Some other SNPs, characterized for EHT by the deletion variant, might participate in the functional impairment of natriuretic peptide system defining an increased genetic susceptibility to EHT [14, 27].

Our findings are in agreement with those of earlier studies demonstrating distribution of the* ANP G191A* genotypes in case and control subjects. Homozygote (GG genotype) displayed higher frequency in case and control subjects of both sexes in comparison to other genotypes. Providing no justification with this astonishing observation, it may be artifactual to some degree because patients were chosen based on blood pressure levels.

## 5. Conclusion

Taken together, earlier single studies were expanded on EHT by proposing that* ANP* gene* I/D* and* G191A* polymorphisms may influence the occurrence of hypertension, particularly in population-based studies. Our observations also keep the inquiry open considering the heterogeneous influence of* ANP* gene* I*/*D* and* G191A* in variant ethnic populations. More functional and genetic studies are guaranteed to clarify the relation between* ANP* gene* I*/*D* and* G191A* polymorphisms and EHT as well as between the* ANP* gene mechanisms and hypertension. The pathophysiological relevance of* ANP *in relation to hypertension-associated phenotypes requires additional studies in various ethnic groups.

## Figures and Tables

**Table 1:** Oligonucleotides for amplification and screening of polymorphisms.

ANP gene polymorphism	Forward and reverse primer	PCR cycling conditions	PCR product (bp)	Reference
A191G	FP 5-AGAGGGGAACCAGAGAGGAACCAG-3 FP 5-CCATCAGGTCTGCGTTGGATAC-3	5 min 95°C 60 sec 55°C 90 sec 72°C 60 sec 94°C ×45 10 sec 72°C 4°C	189 and 197	Kato et al., [26]

I/D	FP 5-GCAGTCCAGCCTAGGTGATA-3 RP 5-TCCGGAGTAGCTAGGACTTACA-3	5 min 95°C 60 sec 55°C 90 sec 72°C 60 sec 94°C ×45 10 sec 72°C 4°C	230 and 238	Kato et al., [26]

PCR cycling conditions are represented as temperature and time of initial denaturation, denaturation, annealing, extension, and final extension × number of cycles.

**Table 2:** Clinical characteristics of EHT, EHT + T2DM, and control subjects.

Parameter	EHT (*N* = 75)	EHT + T2DM (*N* = 88)	Control (*N* = 157)
AGE	59.45 ± 10.34	59.05 ± 11.10	52.51 ± 9.41
SBP	152.01 ± 23.10	150.51 ± 20.00	122.21 ± 11.24
DBP	94.12 ± 10.00	93.00 ± 9.34	76.20 ± 9.00
BMI	26.21 ± 6.02	28.04 ± 4.25	25.00 ± 4.00
FBS	5.46 ± 1.11	7.89 ± 1.35	5.04 ± 0.50
TCH	5.02 ± 1.10	4.53 ± 1.04^**∗**^	5.10 ± 1.31
LDL	3.01 ± 1.10	3.00 ± 1.00	3.21 ± 1.10
HDL	1.34 ± 0.34^*∗*^	1.21 ± 0.30^**∗**^	1.30 ± 0.52
TG	1.50 ± 0.52	2.00 ± 0.53	1.23 ± 0.55

EHT refers to essential hypertensive patients, EHT + T2DM refers to essential hypertensive patients with Type 2 Diabetes Mellitus, and control refers to healthy subjects. *P* value is obtained by the comparisons of means between EHT and control subjects as well as between EHT + T2DM and control subjects, in the same row significant. Value mean ± standard deviation. ^*∗*^Nonsignificant *P* > 0.05.

**Table 3:** Genotypes and allele frequencies distribution of gene polymorphisms between two patient groups and control subjects.

Genotype and allele frequency	EHT	EHT+T2DM	Control
	*n* (%)	*n* (%)	*n* (%)
*Insertion/deletion*			
II	57 (76)	71 (80.7)	133 (84.7)
ID	12 (16)	14 (15.9)	21 (13.4)
DD	6 (8)	3 (3.4)	3 (1.9)
I	126 (84)	156 (88.6)	287 (91.4)
D	12 (16)	20 (11.4)	27 (8.6)
*P* value	0.027^*∗*^/0.015^*∗*^	0.219/0.200	—
*Post hoc test*			
II vs ID	0.749	—	—
II vs DD	0.056	—	—
ID vs DD	0.195	—	—
Odds ratio (95% CI)	0.494 (0.274–0.890)	0.734 (0.399–0.890)	—
*A191G*			
GG	54 (72)	69 (78.4)	124 (79)
GA	17 (22.7)	17 (19.3)	31 (19.7)
AA	4 (5.3)	2 (2.3)	2 (1.3)
G	125 (83.3)	155 (89.1)	279 (88.9)
A	25 (16.7)	19 (10.9)	35 (11.1)
*P* value	0.761/0.067	0.451/0.534	—
Odds ratio (95% CI)	0.627 (0.360–1.093)	1.023 (0.566–1.850)	—

EHT refers to essential hypertensive patients, EHT + T2DM refers to essential hypertensive patients with Type 2 Diabetes Mellitus, and control refers to healthy subjects. ^*∗*^ *P* value < 0.05.

**Table 4:** Genotype analysis based on race.

Race	EHT			EHT + T2DM			Control		
	*N* = 75			*N* = 88			*N* = 157		
	II	ID	DD	II	ID	DD	II	ID	DD
Malay	20 (69.0)	7 (24.1)	2 (6.9)	27 (79.4)	6 (17.6)	1 (2.9)	52 (78.8)	13 (19.7)	1 (1.5)
Chinese	27 (93.1)	0 (0.0)	2 (6.9)	20 (90.9)	1 (4.5)	1 (4.5)	54 (93.1)	3 (5.2)	1 (1.7)
Indian	10 (58.8)	5 (29.4)	2 (11.8)	24 (75.0)	7 (21.9)	1 (3.1)	27 (81.8)	5 (15.2)	1 (3.0)
*P* value		0.033			0.570			0.189	

	GG	GA	AA	GG	GA	AA	GG	GA	AA

Malay	19 (65.5)	9 (31.0)	1 (3.4)	25 (73.5)	8 (23.5)	1 (2.9)	46 (69.7)	20 (30.3)	0 (0.0)
Chinese	26 (89.7)	2 (6.9)	1 (3.4)	20 (90.9)	1 (4.5)	1 (4.5)	52 (89.7)	5 (8.6)	1 (1.7)
Indian	9 (52.9)	6 (35.3)	2 (11.8)	24 (75.0)	8 (25.0)	0 (0.0)	26 (78.8)	6 (18.2)	1 (3.0)
*P* value		0.046			0.264			0.021	

EHT refers to essential hypertensive patients, EHT + T2DM refers to essential hypertensive patients with Type 2 Diabetes Mellitus, and control refers to healthy subjects. *P* value < 0.05.

**Table 5:** Allele frequency analysis based on gender.

Alleles of gene polymorphisms		EHT		EHT + T2DM		Control	
		*N* = 75		*N* = 88		*N* = 157	
		Male	Female	Male	Female	Male	Female
		*N* = 47	*N* = 28	*N* = 43	*N* = 45	*N* = 74	*N* = 83
Insertion/deletion	I	77	49	76	80	132	155
	D	17	7	10	10	16	11
*P* value		0.456		0.551		0.228	
Odds ratio		0.647 (0.250–1.674)		0.950 (0.374–2.410)		0.585 (0.263–1.306)	

A191G	G	77	48	75	80	128	151
	A	17	8	11	10	20	15
*P* value		0.653		0.818		0.215	
Odds ratio		0.755 (0.303–1.884)		852 (0.342–2.123)		0.636 (0.313–1.293)	

EHT refers to essential hypertensive patients, EHT + T2DM refers to essential hypertensive patients with Type 2 Diabetes Mellitus, and control refers to healthy subjects. *P* value > 0.05.
